# Targeting LIMK1 with luteolin inhibits the growth of lung cancer *in vitro* and *in vivo*

**DOI:** 10.1111/jcmm.16568

**Published:** 2021-05-13

**Authors:** Man Zhang, Rui Wang, Jie Tian, Mengqiu Song, Ran Zhao, Kangdong Liu, Feng Zhu, Jung‐Hyun Shim, Zigang Dong, Mee‐Hyun Lee

**Affiliations:** ^1^ China‐US (Henan) Hormel Cancer Institute Zhengzhou China; ^2^ Department of Pathophysiology, School of Basic Medical Sciences Zhengzhou University Zhengzhou China; ^3^ Cancer Research Institute, The Affiliated Hospital of Guilin Medical University Guilin China; ^4^ Department of Pharmacy, College of Pharmacy Mokpo National University Muan Republic of Korea; ^5^ College of Korean Medicine Dongshin University Naju Republic of Korea; ^6^ Present address: Henan Provincial Key Laboratory of Children’s Genetics and Metabolic Diseases Children’s Hospital Affiliated to Zhengzhou University Zhengzhou Henan 450018 China

**Keywords:** LIMK1, luteolin, non‐small cell lung cancer

## Abstract

Lung cancer is the leading cause of cancer‐related deaths. LIM domain kinase (LIMK) 1 is a member of serine/threonine kinase family and highly expressed in various cancers. Luteolin, a polyphenolic plant flavonoid, has been reported to suppress tumour proliferation through inducing apoptosis and autophagy via MAPK activation in glioma. However, the mechanism of luteolin on suppressing lung cancer growth is still unclear. We found that luteolin targeted LIMK1 from the *in silico* screening and significantly inhibited the LIMK1 kinase activity, which was confirmed with pull‐down binding assay and computational docking models. Treatment with luteolin inhibited lung cancer cells anchorage‐independent colony growth and induced apoptosis and cell cycle arrest at G1 phase. Luteolin also decreased the expression of cyclin D1 and increased the levels of cleaved caspase‐3 by down‐regulating LIMK1 signalling related targets, including p‐LIMK and p‐cofilin. Furthermore, luteolin suppressed the lung cancer patient‐derived xenograft tumour growth by decreasing Ki‐67, p‐LIMK and p‐cofilin expression *in vivo*. Taken together, these results provide insight into the mechanism that underlies the anticancer effects of luteolin on lung cancer, which involved in down‐regulation of LIMK1 and its interaction with cofilin. It also provides valuable evidence for translation towards lung cancer clinical trials with luteolin.

## 1 INTRODUCTION

Lung cancer is the most leading cause of both men and women cancer death, and new cases in the worldwide.^1^, ^2^ While there is clearly an established risk for lung cancer associated with cigarette smoking, recent data indicated that increased risk of lung cancer in never smokers, especially in women.^2^ Non‐small cell lung cancer (NSCLC) is a major type (~85%) of lung cancer, and its targeted therapy is tried such as epidermal growth factor receptor tyrosine kinase inhibitors (EGFR‐TKIs); however, the treatment still limited due to acquired resistance and deficient efficacy.^3^ Therefore, we need to continuously develop and investigate proper targets and treatment.

LIM domain kinase (LIMK) 1, contains LIM motifs, PDZ domain and kinase domain, is a serine/threonine kinase and involved in the cell survival, migration and invasion of lung, breast, prostate cancer and leukaemia.^4^ LIMK1 is activated from the upstream kinase such as Rho‐associated coiled‐coil‐containing protein kinase (ROCK) and transfer the signal to downstream effecter, cofilin by phosphorylation thereby promotes cancer cell growth through regulation of actin/filament dynamics.

Luteolin, a dietary flavone derived from vegetables, fruits and herbs, traditionally used in Chinese medicine.^5^, ^6^ It has beneficial effects including anti‐inflammatory, anti‐allergic, anticancer and antioxidant, to prevent the diseases.^5^, ^6^, ^7^, ^8^, ^9^ Compared with traditional chemotherapy drug, luteolin showed less toxicity as a natural compound.^10^, ^11^ It inhibits critical events associated with carcinogenesis, including cell invasion, metastasis, transformation and angiogenesis, by inhibiting transcription factors, kinase modification and cell cycle arrest and inducing apoptosis.^5^, ^7^, ^9^, ^10^ Zao et al demonstrated that luteolin epigenetically activated the Nrf2 pathway by down‐regulating DNA methyltransferase (DNMT) and histone deacetylase (HDAC) expression.^11^ Kang et al demonstrated that luteolin promoted apoptotic human colon cancer cells death by up‐regulation of Nrf2 expression through inhibition of DNA demethylase and the interaction of Nrf2 with p53.^5^ The anticancer effect of luteolin has been investigated in various cancers including cervical, gastric, lung, colon and breast cancer.^12^, ^13^, ^14^, ^15^, ^16^, ^17^ Aneknan et al showed that luteolin inhibited the proliferation of human cholangiocarcinoma cells by inducing apoptosis and interrupting the JNK/STAT3 pathway.^12^ Also You et al mentioned that luteolin suppresses tumour proliferation through inducing apoptosis and autophagy via MAPK activation in glioma.^18^ However, it is not known whether luteolin regulates signalling protein kinase in lung cancer, and this knowledge might be important in exploring its favourable anticancer effect in lung cancer.

This study aims to investigate the anticancer effects of luteolin on human lung cancer cells by targeting LIMK1. Our findings show that luteolin inhibits cell proliferation in lung cancer cells and induces cell cycle arrest and apoptosis. These activities are mediated by inhibition of LIMK1 and related signalling pathway.

## 2 MATERIALS AND METHODS

### 2.1 Reagents

Luteolin (purity ≥ 98%) was purchased from Sichuan Weikeqi Biological Technology CO., Ltd (CAS#: 491‐70‐3). BMS‐5 (purity ≥ 98%) was purchased from Enzo Life Sciences (CAS#:1338247‐35‐0). CNBr‐activated Sepharose^TM^ 4B (Lot# 10265330) was purchased from GE Healthcare. Thiazolyl blue tetrazolium bromide (MTT) powder was purchased from Solarbio Technology Co., Ltd. Dimethylsulfoxide (DMSO) was purchased from Sigma. Cyclin D1 (catalog# 2922), cyclin D3 (catalog# 2936), Bax (catalog# 5023), cleaved‐PARP (catalog# 5625), caspase‐3 (catalog# 9662), cleaved caspase‐3 (catalog# 9664), caspase‐7 (catalog# 9492), cleaved caspase‐7 (catalog# 8438), ROCK1 (catalog# 4035) and ROCK2 (catalog# 9029) were purchased from Cell Signaling Technology. Phospho‐LIMK‐1/2 (Thr 508/505)‐R (catalog# sc‐28409‐R), LIMK‐1(catalog# sc‐28370), cofilin (catalog# sc‐33779) and phospho‐cofilin (mSer3)‐R (catalog# sc‐21867‐R) were purchased from Santa Cruz Technology.

### 2.2 Cell cultures

The human normal lung epithelial cell (NL‐20) and lung cancer cell lines (NCI‐H1975 and NCI‐H1650) were purchased from American Type Culture Collection (ATCC). Normal lung cell NL‐20 was cultured in Ham's F12 medium with 1.5 g/L sodium bicarbonate, 2.7 g/L glucose, 2.0 mmol/L L‐glutamine, 0.1 mmol/L nonessential amino acids, 0.005 mg/ml insulin, 10 ng/mL epidermal growth factor, 0.001 mg/mL transferrin, 500 ng/mL hydrocortisone and 4% foetal bovine serum. NCI‐H1975 and NCI‐H1650 cells were cultured in RPMI‐1640 containing penicillin (100 units/mL), streptomycin (100 μg/mL) and 10% foetal bovine serum (Biological Industries, Israel) and grown at 37°C in a humidified incubator containing 5% CO_2_. All cells were cytogenetically tested and authenticated before being frozen. Each vial of frozen cells was thawed and maintained in culture for a maximum of 2 months.

### 2.3 Cell proliferation assay

Logarithmic phase cells were collected and seeded (about 2000‐7000 cells per well) in 96‐well plates and then incubate 24 hours prior to treatment with different doses of luteolin. After 24, 48 and 72 hours incubation, 20 μL of MTT (5 mg/mL) reagent were added in each well. One hour later, the supernatant was discarded carefully and 100 μL of DMSO were added in each well to dissolve the crystallized formazan, then absorbance was measured at 570 nm. Six replicate wells were used for each compound concentration.

### 2.4 Anchorage‐independent cell growth assay

NCI‐H1975 and NCI‐H1650 cells were suspended in complete growth media and cell concentration were adjusted to 8,000 per millilitre, and then mixed with 0.3% agar contained different doses of luteolin was added in a top layer up a base layer of 0.5% agar in 6 well plates. The colonies were grown at 37°C in a humidified incubator containing 5% CO_2_. Two or three weeks later, the cell colonies were counted and photographed under a microscope using the Image‐Pro Plus software (v.6.0) program (MediaCybernetics).

### 2.5 Cell cycle and apoptosis analysis

Cells were seeded in 60‐mm plates and cultured overnight at 37°C in a 5% CO_2_ incubator and then treated with 0, 5, 10, 20 or 40 μmol/L of luteolin for 48 or 72 hours. For cell cycle analysis, cells were then fixed in 70% ethanol and stored at ‐20°C for 24 hours. After staining with annexin‐V for apoptosis or propidium iodide for cell cycle assessment, cell cycle contribution and apoptotic cells were analysed using a BD FACSCalibur Flow Cytometer (BD Biosciences).

### 2.6 Western blot analysis

Cell lysates were obtained from cultured lung cancer cells treated with DMSO or luteolin and were prepared in RIPA buffer (150 mM NaCl, 0.5–1% NP‐40, 50 mM Tris–HCl, with 1 mM PMSF and protease inhibitor mixture). 8%‐15% SDS‐polyacrylamide gel electrophoresis was used and transferred to polyvinylidene fluoride membranes then membranes were blocked with 5% skim milk in PBS. Primary antibodies, ROCK1, ROCK2, p‐LIMK (Thr508/505), LIMK1, phospho‐cofilin (p‐cofilin) and cofilin were incubated at 4°C, overnight. Blots were washed 3 times in 1×PBST buffer and followed by incubation with the appropriate horseradish peroxidase (HRP)‐linked IgG. Blots were visualized by the enhanced chemiluminescence (ECL) detection reagent (GE Healthcare Life Science, Little Chalfont, HP, UK) and Amersham Image 600 imager (GE, Milwaukee, WI, USA).

### 2.7 *In vitro* and *ex vivo* pull‐down assays

NCI‐H1975 cell lysates (500 μg) were incubated with luteolin‐Sepharose 4B (or Sepharose 4B only as a control) beads in reaction buffer (50 mmol/L Tris pH 7.5, 5 mmol/L EDTA, 150 mmol/L NaCl, 1 mM DTT, 0.01% NP‐40 and 2 mg/mL bovine serum albumin). After incubation with gentle shaking overnight at 4°C, the beads were washed 3 times with washing buffer (50 mmol/L Tris, pH 7.5, 5 mmol/L EDTA, 150 mmol/L NaCl, 1 mmol/L DTT and 0.01% NP‐40), and binding was visualized by Western blotting.

### 2.8 Computational docking model

In order to confirm whether luteolin can bind with LIMK1 kinase, we performed *in* *silico* docking method by using the Schrödinger Suite 2016 software programs (Schrödinger, 2016).^19^ The LIMK1 structure was built with Prime followed by refining and minimizing loops in the binding site. The structure was prepared under the standard methods of the Protein Preparation Wizard (Schrödinger Suite 2016). Hydrogen atoms were added maintained pH to 7, and all water molecules were discarded. The LIMK1 ATP‐binding site based receptor grid was generated for docking. Hence, we can get luteolin computational docking site.

### 2.9 Patient‐derived xenograft mouse model

6 to 8‐week‐old female SCID mice were used in our PDX animal experiments. Qi et al demonstrated that the gender of SCID mice did not have a major impact on animal model development nor drug responses in vivo, and SCID mice of both genders are appropriate for use.^20^ This study was approved by the Ethics Committee of Zhengzhou University (Zhengzhou, Henan, China). Patient‐derived tumour was divided in same mess and then implanted subcutaneously into the back of mice. When average tumour volume was reached about 100 mm^3^ and then divided into two groups. (1) Vehicle group (n = 7); (2) 100 mg/kg dose of luteolin (n = 7).^21^ Luteolin was administered by gavage every day, and tumour volume was measured twice a week. Tumour volume was calculated by following formula: tumour volume (mm^3^) = (length × width × height × 0.52). When average tumour volume was reached about 1000 mm^3^, mice were euthanized and tumours were extracted to further analysis.

### 2.10 Immunohistochemistry (IHC) assay

Paraffin‐embedded tumour tissues were prepared for H&E staining and IHC analysis. When antigen unmask finished, the tumour tissues were blocked with 5% goat serum and incubated at 4°C overnight with antibodies to detect some protein markers, such as Ki‐67, p‐LIMK (Thr508/505) and p‐cofilin. After incubation with a rabbit secondary antibody, DAB (3,3'‐diaminobenzidine) staining was used to visualize the protein targets according to the manufacturer’s instructions. Sectioned tissues were counterstained with haematoxylin, dehydrated through a graded series of alcohol into xylene and mounted under glass coverslips. After then, photographed under a microscope and analysed using the Image‐Pro Plus software (v.6.0) program (MediaCybernetics).

### 2.11 Statistical analysis

Data illustrated with error bars are the mean ± SD. Statistically significant differences were determined using the Student’s *t* test or one‐way ANOVA. For all analysis, a *p* value less than 0.05 was considered statistically significant.

## 3 RESULTS

### 3.1 LIMK is a potential target in lung cancer cells

**FIGURE 1:**
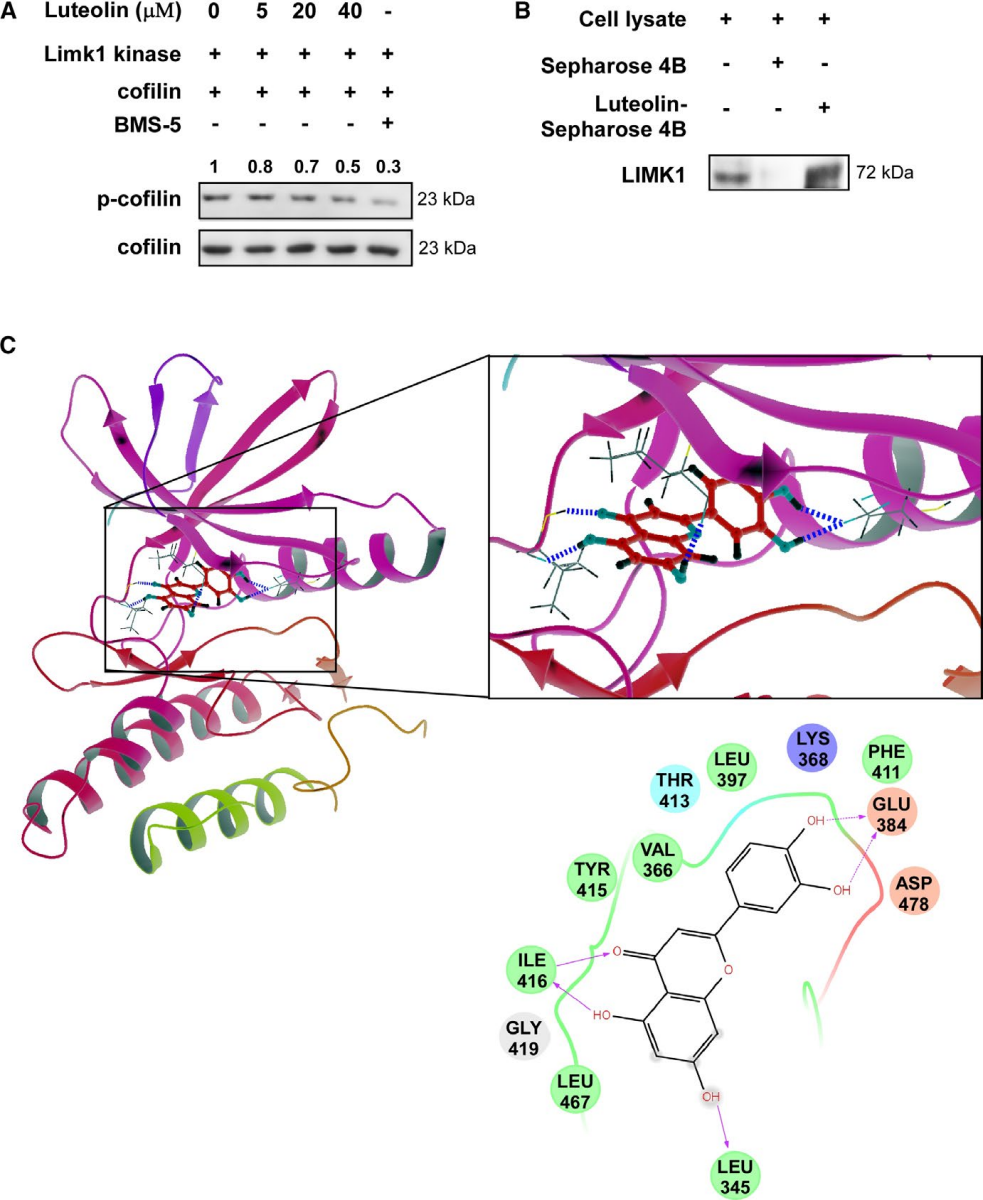
LIMK1 is a potential target of luteolin. (A) Luteolin inhibits LIMK1 kinase activity and down‐regulates the phosphorylation of the LIMK1 substrate p‐cofilin. LIMK1 inhibitor BMS‐5 used as positive control. The bands were quantitated by image J (NIH). (B) The binding of luteolin to LIMK1 in H1975 cell lysates was determined using Sepharose 4B or luteolin‐conjugated Sepharose 4B beads. (C) The interaction between luteolin and LIMK1 was predicted using a computational docking model

To targeting the LIMK1 kinase, we performed *in silico* virtual screening and found luteolin as a candidate. Then, we implemented an *in vitro* kinase assay with active LIMK1 in the presence of 5, 20 or 40 μmol/L luteolin and results showed that the phosphorylation of cofilin, a LIMK1 substrate, was decreased by treatment with luteolin compared with DMSO control (Figure 1A). LIMK1 inhibitor BMS‐5 was used as positive control. Luteolin suppressed the kinase activity of LIMK1 in a dose‐dependent manner. To verify the target of luteolin, we examined *ex vivo* pull‐down binding assays. Sepharose 4B beads conjugated with luteolin, but not Sepharose 4B beads only, bound with LIMK1 in NCI‐H1975 cell lysates (Figure 1B). Computational docking analysis results ascertained the direct interaction between luteolin and LIMK1 (Figure 1C). Luteolin interacted with leucine (LEU) 345, glutamic acid (GLU) 384 and isoleucine (ILE) 416 of LIMK1 kinase by hydrogen bond. These results indicated that luteolin might be a potential effective inhibitor of LIMK1.

### 3.2 Luteolin inhibits the proliferation of lung cancer cells

**FIGURE 2:**
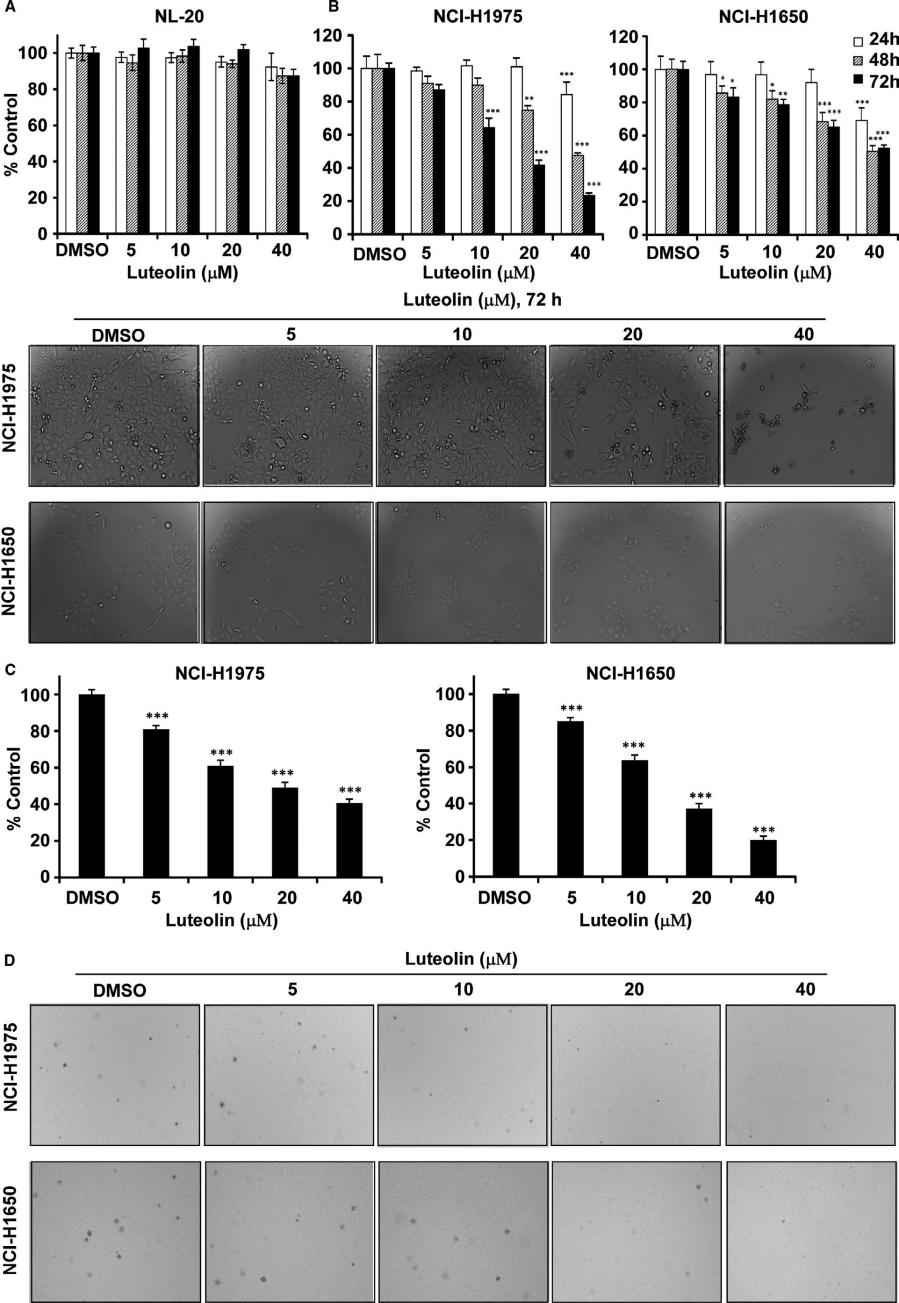
Luteolin inhibits the growth of lung cancer cells. (A) Cytotoxic effect of luteolin on normal lung cell, NL‐20. (B) Growth effects of luteolin (5, 10, 20 and 40 µM) on NCI‐H1975 and NCI‐H1650 lung cancer cells. Proliferation of cells was estimated by MTT assay at 24, 48 or 72 h. Data were shown compared with DMSO treated group. *P < .05; **P < .01; ***P < .001 compared with controls. (C) Anchorage‐independent cell growth effect of luteolin on NCI‐H1975 and NCI‐H1650 lung cancer cells. Colonies were captured and the number was counted after 2‐3 weeks, the results were presented as treated group compared with control group. ***P < .001. (D) Representative photographs of anchorage‐independent cell growth assay results of cells treated or not treated with luteolin

The potency of luteolin on the cytotoxicity and proliferation of normal lung cells and lung cancer cells were determined by MTT assay (Figure 2). We first investigated the effect of luteolin on the proliferation of normal human NL‐20 lung cells. Incubation of NL‐20 cells with luteolin (5, 10, 20 or 40 μmol/L) for 24, 48 or 72 hours showed that highest concentration (40 μmol/L) did not affect toxicity (Figure 2A). Therefore, we selected a maximal nontoxic concentration (40 μmol/L) of luteolin for further experiments. Treatment of NCI‐H1975 and NCI‐H1650 lung cancer cells with luteolin (20 or 40 μmol/L) significantly inhibited the proliferation in a time‐dependent manner compared with a DMSO control (Figure 2B). Luteolin also attenuated anchorage‐independent cell growth of these two lung cancer cell lines in a concentration‐dependent manner compared with DMSO treated control (Figure 2C,D). Representative images of colonies illustrate the number of colonies (Figure 2D).

### 3.3 Luteolin directly inhibits LIMK1 activity

**FIGURE 3:**
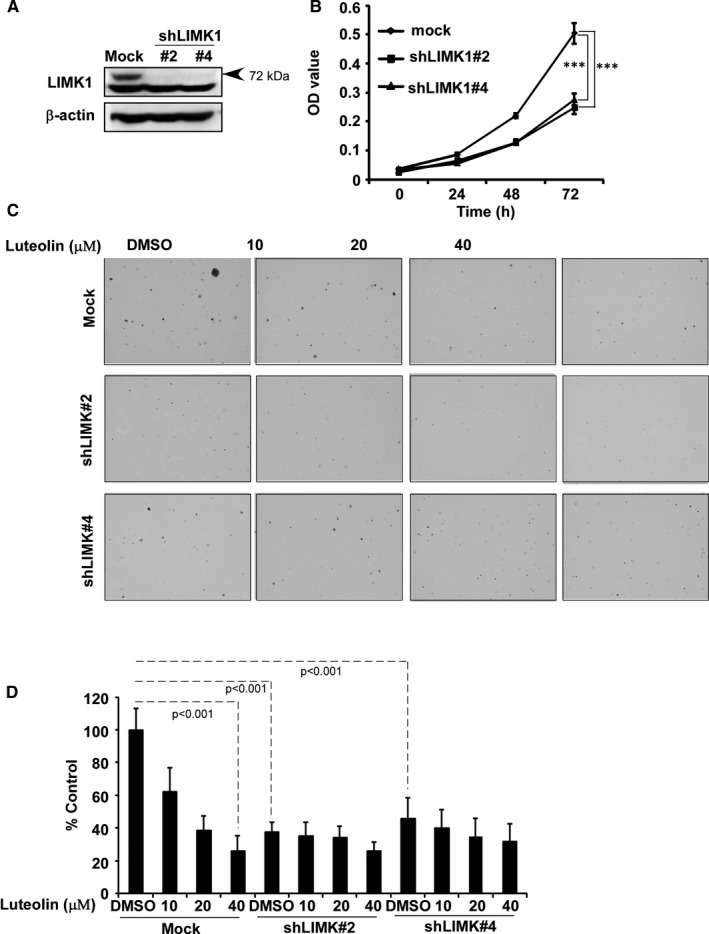
LIMK1 is a potential target of luteolin. (A) The expression of LIMK1 in NCI‐H1975 cells after knocking‐down LIMK by Western blot. (B) Proliferation of cells expression shmock and shLIMK #2 and 4 were examined by MTT. The asterisks (*P < .05, **P < .01, ***P < .001) indicate a significant decrease in proliferation compared with corresponding control. (C) Anchorage‐independent growth was assessed after treatment with serially doses of luteolin in NCI‐H1975 cells expressing shmock and shLIMK #2 and 4. The asterisks (*P < .05, **P < .01, ***P < .001) indicate a significant decrease in colony number compared with corresponding control. Data are shown as mean ± SD of values from triplicate samples

To confirm the role of luteolin in inhibiting LIMK activity, we performed LIMK knockdown cells by infecting NCI‐H1975 cells with virus particles containing shmock or shLIMK (Figure 3) and then conducted the proliferation of cells by MTT assay and the anchorage‐independent cell growth assay (Figure 3B‐D). The results showed that the proliferation rate of cells significantly decrease after knockdown of LIMK (Figure 3B). The colonies number of shLIMK contained cells were decreased compared with shmock transfected cells and luteolin did not much influence in shLIMK cells compared with shmock cells (Figure 3C,D).

### 3.4 Luteolin induces cell cycle arrest and apoptosis of lung cancer cells

**FIGURE 4:**
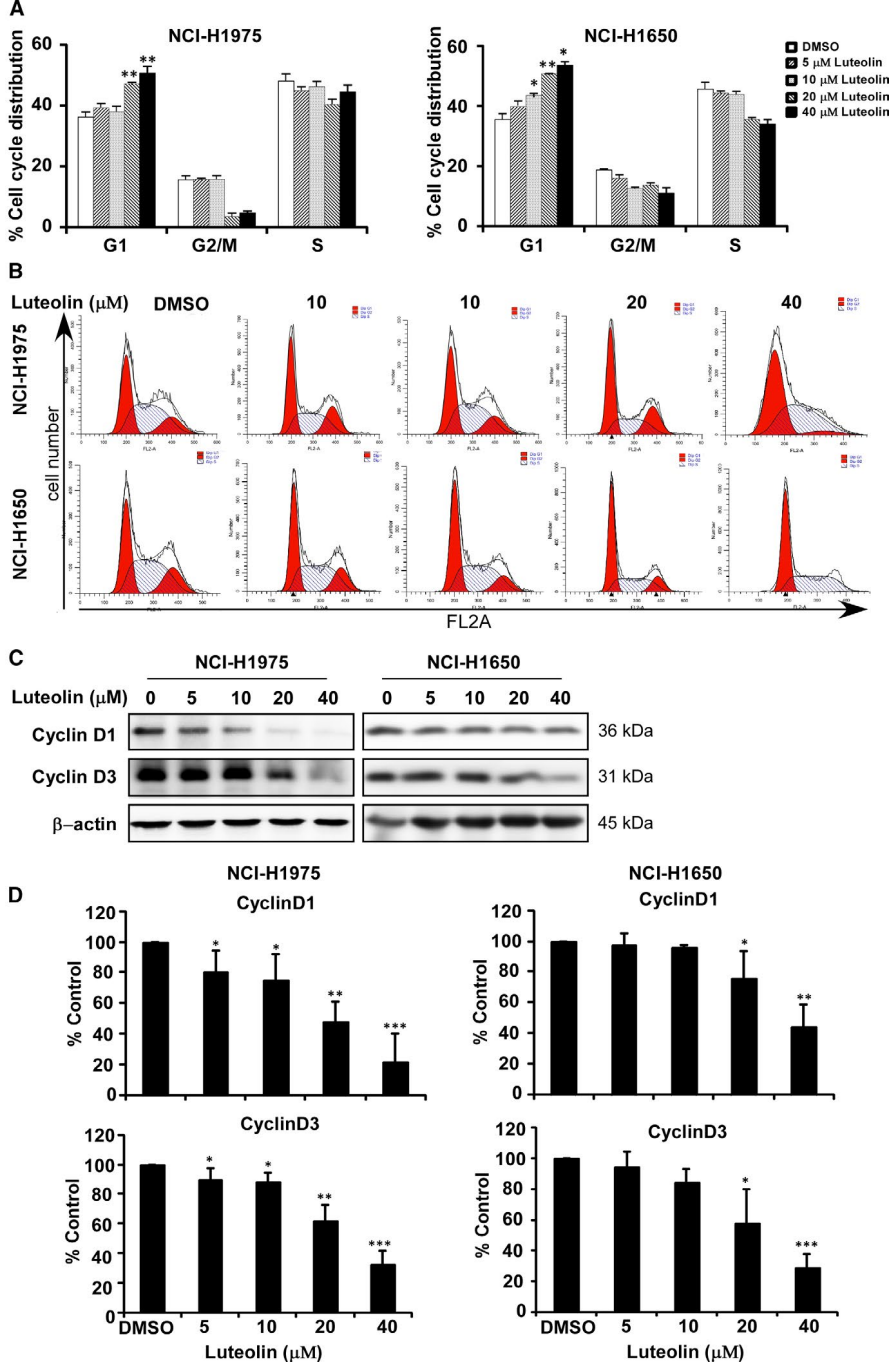
Luteolin induces cell cycle arrest and apoptosis of lung cancer cells. Effects of luteolin on cell cycle were in NCI‐H1975 and NCI‐H1650 lung cancer cells (A). Cells were treated with DMSO or 5, 10, 20 and 40 μmol/L of luteolin and then incubated for 48 h (cell cycle analysis, cell cycle marker expression). Representative plots of flow cytometry analysis of cell cycle of NCI‐H1975 and NCI‐H1650 (B). Data were shown compared with DMSO treated group. *P < .05; **P < .01; ***P < .001 compared with controls. The effects of luteolin on the expression of biomarkers associated with cell cycle (cyclin D1 and D3) (C) are shown by Western blot. Quantitative analysis results of three bathes of biomarkers associated with cell cycle (cyclin D1 and D3) (D)

**FIGURE 5:**
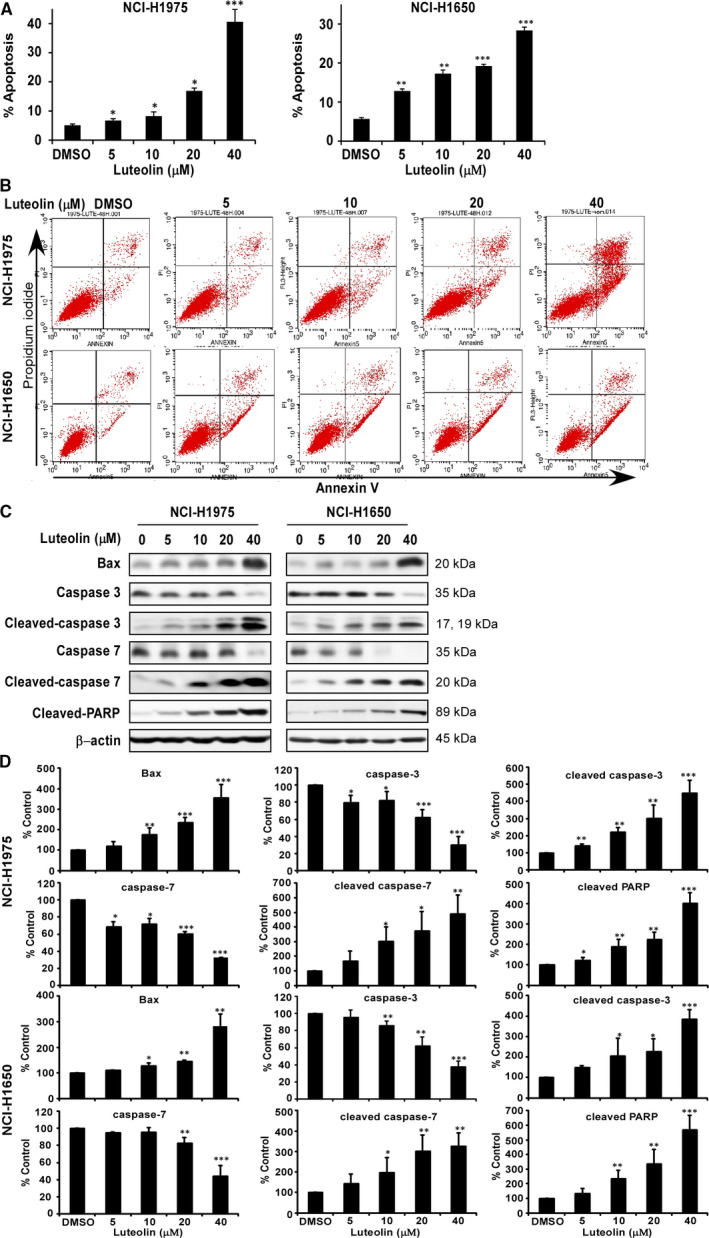
Luteolin induces cell apoptosis of lung cancer cells. Effects of luteolin on cell apoptosis were in NCI‐H1975 and NCI‐H1650 lung cancer cells (A). Cells were treated with DMSO or 5, 10, 20 and 40 μmol/L of luteolin and then incubated for 72 h (annexin‐V staining assay, apoptosis marker expression). Representative plots of flow cytometry analysis of cell apoptosis of NCI‐H1975 and NCI‐H1650 (B). Data were shown compared with DMSO treated group. *P < .05; **P < .01; ***P < .001 compared with controls. The effects of luteolin on the expression of biomarkers associated with cell apoptosis (Bax, caspase‐3, cleaved caspase‐3, caspase‐7, cleaved caspase‐7 and cleaved PARP) (C) are shown by Western blot. Quantitative analysis results of three bathes of biomarkers associated with cell apoptosis (Bax, caspase‐3, cleaved caspase‐3, caspase‐7, cleaved caspase‐7 and cleaved PARP) (D)

To examine whether the cell growth inhibition by luteolin was from the regulation of cell cycle and apoptosis, we analysed cell cycle contribution and annexin V staining cells (Figure 4). The results revealed that luteolin‐induced cell cycle arrest at G1 phase in NCI‐H1975 and NCI‐H1650 (Figure 4A,B). To apoptosis analysis, NCI‐H1975 and NCI‐H1650 cells were treated with DMSO control and luteolin at the concentration of 5, 10, 20 or 40 μmol/L for 72 hours. Luteolin significantly induced apoptosis in a dose‐dependent manner (Figure 5A,B). Based on the effects induced by luteolin, we examined the expression of proteins associated with the G1 phase of cell cycle and apoptosis by Western blot (Figure 4C,D). Treatment of lung cancer cells with luteolin decreased the expression of cyclin D1 and cyclin D3, cell cycle markers compared with control (Figure 4C,D). Furthermore, luteolin‐induced apoptosis markers, Bax, cleaved caspase 3, cleaved caspase‐7 and cleaved PARP expression while reduced total form of caspase‐3 and caspase‐7 expression compared with DMSO controls, respectively (Figure 5C,D).

### 3.5 Luteolin down‐regulates LIMK1 signalling pathways

**FIGURE 6:**
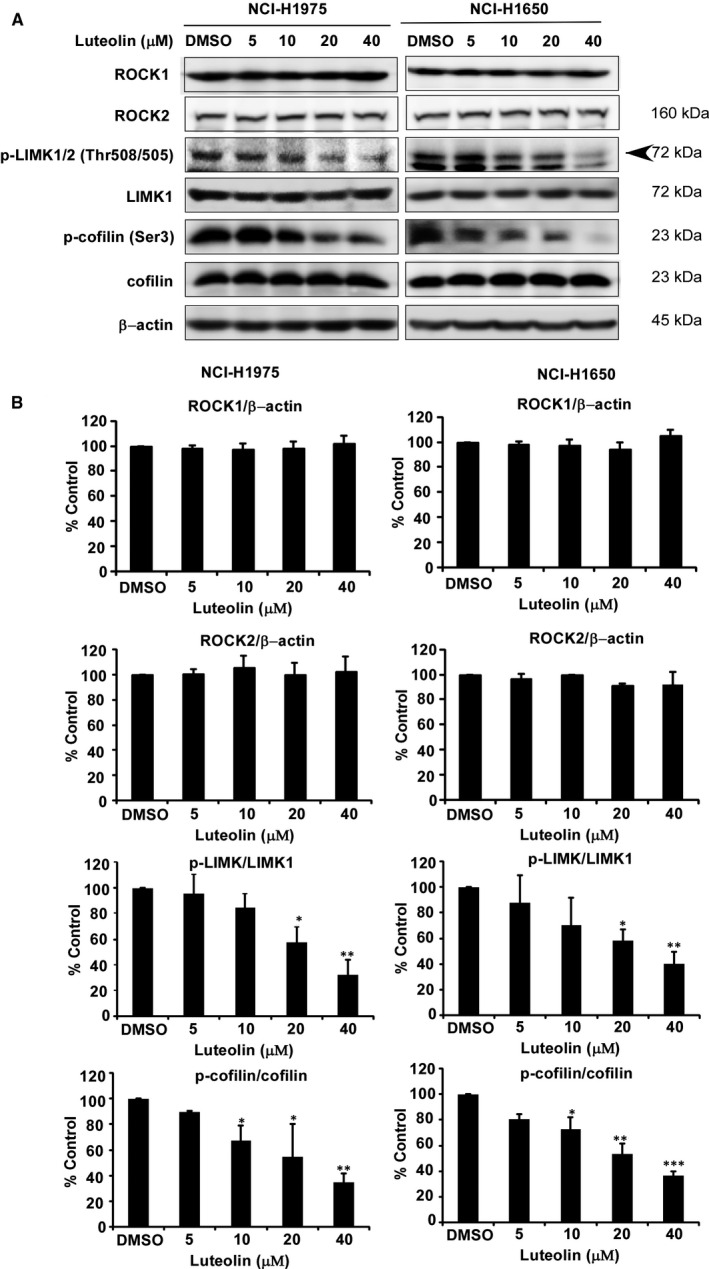
Luteolin down‐regulates LIMK1‐related signalling pathways. NCI‐H1975 and NCI‐H1650 cells were treated with increasing doses of luteolin (5, 10, 20 and 40 μmol/L). The expression and /or phosphorylation of the indicated proteins, ROCK1, ROCK2, p‐LIMK, LIMK1, p‐cofilin and cofilin were assessed by Western blot (A). β‐Actin was used for the internal control to verify equal protein loading. Quantitative analysis results of three bathes of indicated proteins associated with LIMK1‐related signalling pathways, ROCK1, ROCK2, p‐LIMK, LIMK1, p‐cofilin and cofilin (B). Data were shown compared with DMSO treated group. *P < .05; **P < .01; ***P < .001 compared with controls

To identify the effect of luteolin on LIMK1 signalling pathways, we treated the NCI‐H1975 and NCI‐H1650 cells with luteolin and detected their expressions (Figure 6). Luteolin decreased the levels of phosphorylated p‐LIMK1/2 and p‐cofilin compared with total form of LIMK1, cofilin or DMSO treated controls, whereas ROCK1 and ROCK2 not changed (Figure 6A,B).

### 3.6 Luteolin suppresses LIMK1‐mediated tumour growth in patient‐derived xenograft mice

**FIGURE 7:**
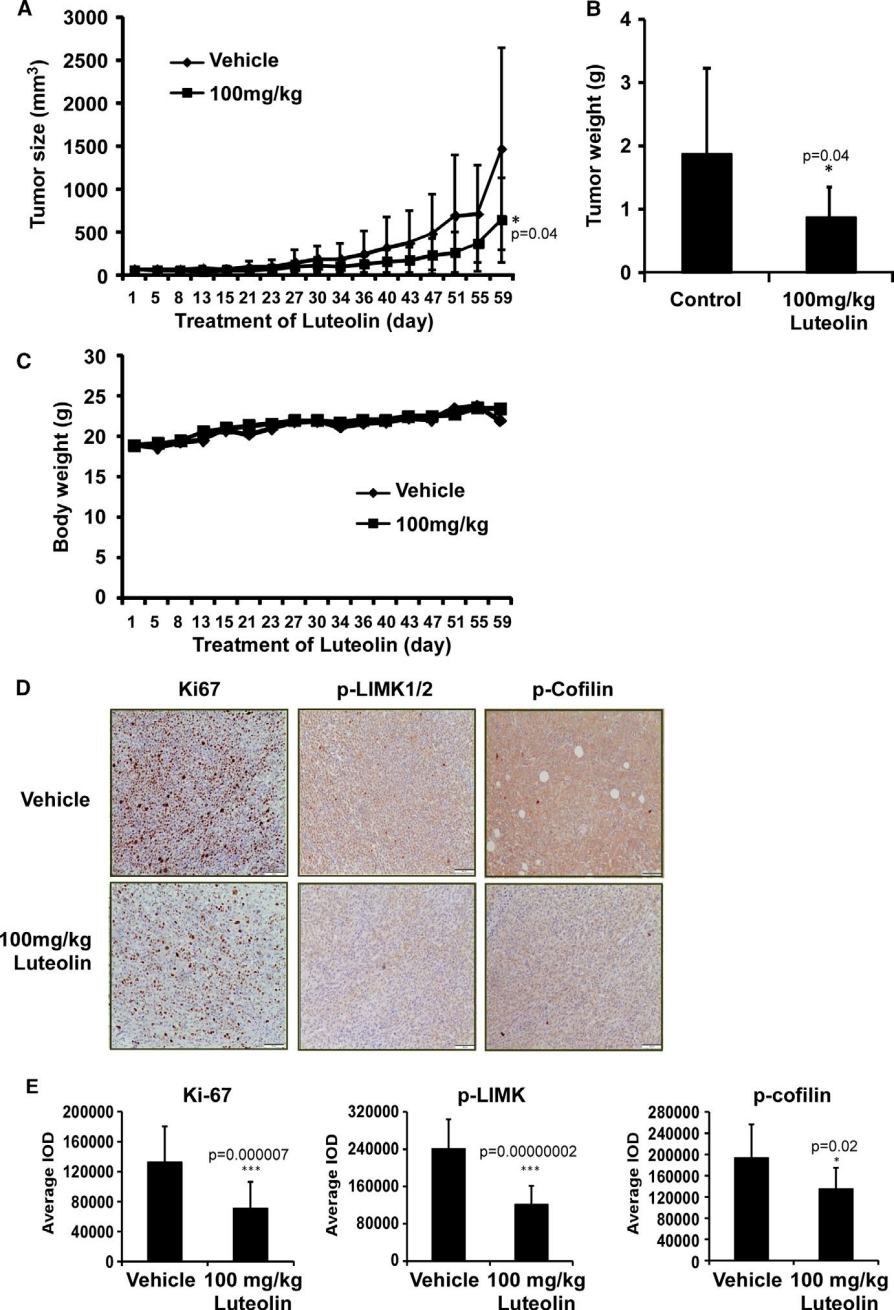
Luteolin attenuates the growth of PDX tumours in mice. (A) The effect of luteolin on the volume of PDX tumours was plotted over 59 days. Vehicle or 100 mg/kg luteolin was administered by gavage. Tumour volume was measured twice a week. (B) The tumour weight was measured when the mice were sacrificed. The asterisk (*P < .05) indicates a significant decrease in volume and weight of tumours from vehicle and luteolin‐treated mice. Data are shown as mean values ± SD. (C) Body weight of mice was plotted over 59 days. (D) Representative photographs of Ki‐67, p‐LIMK and p‐cofilin expression. The expressions were examined by IHC analysis (100× magnification). (E) Quantitation graph of Ki‐67, p‐LIMK and p‐cofilin expression. The expressions were quantified from 4 separate areas on each slide and an average of 5 (vehicle) or 5 (luteolin‐treated) samples per group. Data are expressed as IOD values ± SD. The asterisks (*P < .05, **P < .01, ***P < .001) indicate a significant decrease in Ki‐67, p‐LIMK and p‐cofilin in treated tissues compared with vehicle‐treated controls

The anticancer activity of luteolin was then evaluated in lung cancer patient‐derived xenograft (PDX) mice experiments (Figure 7 and supplementary Figure S3). Everyday administration of luteolin (100 mg/kg) for 59 days retarded the tumour growth and weight without changing mouse body weight compared with vehicle‐treated control (Figure 7A‐C and supplementary Figure 3A‐B). To determine whether the correlation between luteolin efficacy and Ki‐67, p‐Limk1/2 and p‐cofilin expression that we observed *in vitro* could be recapitulated *in vivo*, we performed immunohistochemistry (IHC) analysis in the tumour samples (Figure 7D,E). The results showed that proliferation marker Ki‐67 and signalling marker p‐Limk1/2 and p‐cofilin expression were significantly decreased in luteolin‐treated tissues compared with vehicle treated (Figure 7D,E and supplementary Figure S3C). Therefore, we reinforced the notion that p‐Limk1/2 expression is essential for the anticancer activity of luteolin.

## 4 DISCUSSION

Natural compounds from plants have drawn more and more attention due to their function in protecting and suppressing the growth of different human cancers. These food‐based products, being chemo‐preventive agents, are considered to be safer and more effectual against proliferation of cancer.^22^ Luteolin is a flavonoid found in different plants such as vegetables, medicinal herbs and fruits.^5^, ^6^ It acts as an anticancer agent against various types of human malignancies such as lung, breast, prostate, colon and pancreatic cancer through apoptosis induction, cell cycle arrest and proliferation inhibition as well as metastasis down‐regulation.^8^, ^9^ However, the molecular mechanisms of its anticancer activities are still unclear. In this study, we found that luteolin has the potential to treat lung cancer due to its ability to regulate a new target LIMK1/cofilin signalling pathway. As activation of LIMK/cofilin signalling induces cancer development, invasion and metastasis, it is certain that intervening expression and activity can retard cancer cell proliferation, migration and invasion through modulation the target gene expression.^23^, ^24^, ^25^, ^26^ Herein, we investigated LIMK inhibitor from the Chinese medicine library by *in silico* virtual screening and selected luteolin as a candidate. Then, we confirmed the binding between luteolin and LIMK1 and inhibition of kinase activity by luteolin (Figure 1), whereas luteolin did not affect the mRNA level of *limk* (Supplementary Figure S1). Byun et al reported that luteolin targeted to protein kinase Cε (PKCε) and Src kinase activities and inhibited UVB‐induced skin carcinogenesis and its signalling pathways.^27^ The inhibitory activity of luteolin on PKCε and Src kinases was similar against LIMK kinase activity at 20 µmol/L (Figure 1A). In this study, we have used LIMK highly expressed lung cancer cells, NCI‐H1975 and NCI‐H1650. When we knockdown of LIMK expression in Figure 3, the anchorage‐independent colony number was significantly decreased and luteolin could not do further inhibition. It means LIMK is the major target of luteolin. After then, luteolin decreased the expression of p‐LIMK and its downstream p‐cofilin in a dose‐dependent manner (Figure 6). Treatment with luteolin effectively inhibited cancer characteristic phenotypes of NCI‐H1975 and NCI‐H1650 such as cells proliferation and colony formation as well as G1 phase arrest of cell cycle and apoptosis induction in a dose‐dependent fashion (Figure 4A‐D, 5A‐D). Furthermore, the cell cycle and apoptosis marker, cyclin D1, D3 and Bax, cleaved caspase‐3 and cleaved caspase‐7 expression had significantly regulated by treatment of luteolin (Figure 4C, 5C). In this study, a significant inhibition effect of luteolin was performed in PDX mice model, which is an effective preclinical model reflecting human tumour growth.^28^, ^29^ Luteolin inhibited the tumour growth *in vivo,* which contained high expression of LIMK. With the deep understanding of the LIMK/cofilin signalling pathway, many studies have focussed on the inhibitors of LIMK. BMS‐3 and BMS‐5, the thiazolyl amide derivatives, have shown high inhibition activities against LIMK1. They can inhibit the proliferation of mouse 4T1.2 and human MDA‐MB‐231 breast cancer cells.^30^ The cell growth inhibitory effect of BMS‐5, with a concentration of 10 μmol/L, is similar luteolin with a concentration of 20 μmol/L (supplementary Figure S2). CRT0105446 and CRT0105950 can reduce MCF‐7 breast cancer cell growth and MDA‐MB‐231 cell invasion by decreasing expression of phosphorylated cofilin.^31^, ^32^ Damnacanthal, a major component of morinda citrifolia, can inhibit migration and invasion of MDA‐MB‐231 breast carcinoma cells by suppressing phosphorylated cofilin.^33^ Cucurbitacin I and E can inhibit kinetic activity of LIMK to phosphorylate cofilin and then retarded proliferation and migration of HeLa cells and Caco‐2 human epithelial colorectal adenocarcinoma.^34^, ^35^ Though so many LIMK1 inhibitors exist, few inhibitors act on non‐small cell lung adenocarcinoma cells.

Natural compound, diallyl disulfide from garlic extract also reduced the colorectal cancer cell migration and invasion^35^, ^36^. However, still it needs to expand more animal experiments and pharmacokinetics analysis for applying the cancer patient treatment.

In summary, our study identified luteolin as a LIMK inhibitor that suppressed tumour growth by inhibiting LIMK kinase activity and related signalling pathways.

## CONFLICT OF INTEREST

No potential conflicts of interest were disclosed.

## AUTHOR CONTRIBUTION

M. Z, R.W., M. L. and Z. D: conceptualization and design; acquisition of data; analysis and interpretation of data; manuscript drafting. M. Z., R. W and J. T.: experiment performance. M. S., R. Z., K. L. and H. C: data analysis and materials. M.Z., R.W., M. L. and Z. D: manuscript writing. Z. D and M. L: supervision of all study. All authors read and approved the final manuscript.

## Supporting information

Fig S1

Fig S2

Fig S3

## Data Availability

The data that support the findings of this study are available from the corresponding author upon reasonable request.
