# Supplementary figures and images for: Targeting LIMK1 with luteolin inhibits the growth of lung cancer in vitro and in vivo
Source: J Cell Mol Med. 2021 May 13;25(12):5560–71. doi: 10.1111/jcmm.16568 (PMC8184676; doi:10.1111/jcmm.16568)

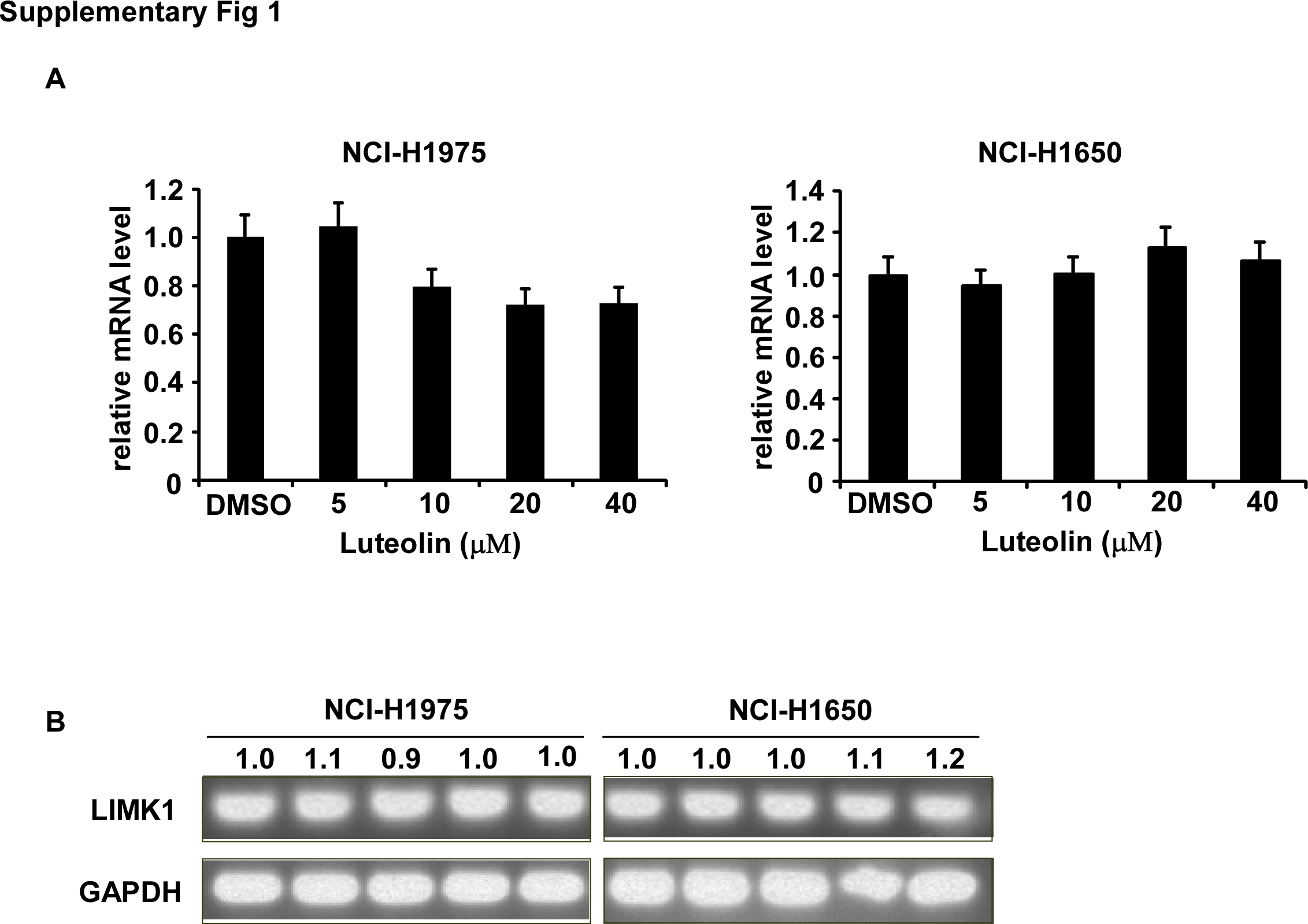

Supplement: Supplementary file 1 — Fig S1 [file JCMM-25-5560-s001.jpg]

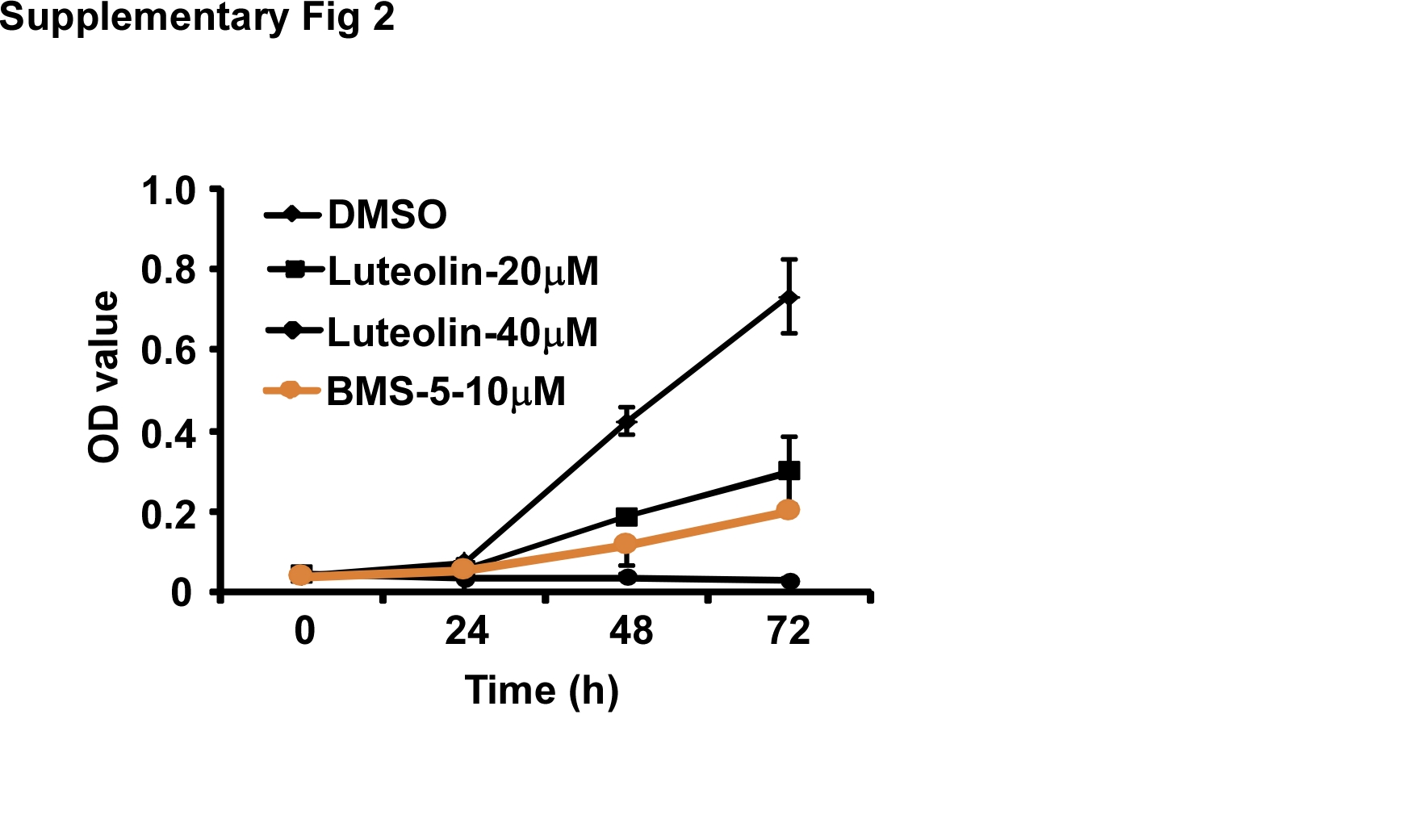

Supplement: Supplementary file 2 — Fig S2 [file JCMM-25-5560-s003.jpg]

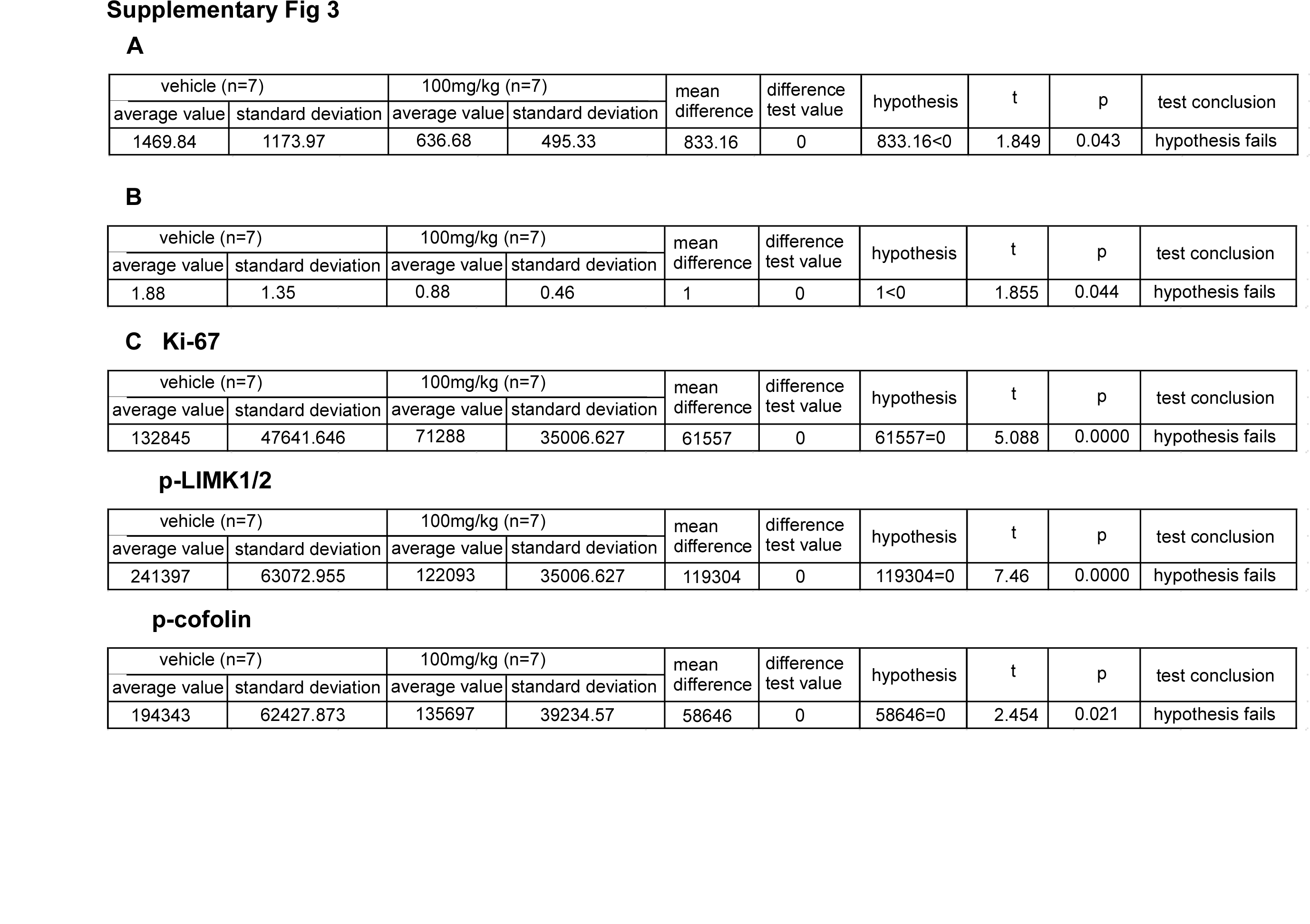

Supplement: Supplementary file 3 — Fig S3 [file JCMM-25-5560-s002.jpg]
